# Effects of differences in femoral anteversion and hip flexion angle on hip abductor muscles activity during clam exercise in females

**DOI:** 10.1371/journal.pone.0305515

**Published:** 2024-06-24

**Authors:** Sho Mitomo, Junya Aizawa, Kenji Hirohata, Takehiro Ohmi, Shunsuke Ohji, Hidetaka Furuya, Tomoko Kawasaki, Yayoi Sakai, Kazuyoshi Yagishita, Atsushi Okawa

**Affiliations:** 1 Department of Orthopaedic and Spinal Surgery, Graduate School of Medical and Dental Sciences, Tokyo Medical and Dental University, Tokyo, Japan; 2 Sports for Health Division, Japan Sports Agency, Tokyo, Japan; 3 Faculty of Health Science, Department of Physical Therapy, Juntendo University, Tokyo, Japan; 4 Clinical Center for Sports Medicine and Dentistry, Tokyo Medical and Dental University, Tokyo, Japan; 5 Department of Rehabilitation, Sonoda Third Hospital, Tokyo, Japan; 6 Department of Rehabilitation, Sonoda Medical Institute Tokyo Spine Center, Tokyo, Japan; 7 Department of Rehabilitation, Kawakita General Hospital, Tokyo, Japan; Universitatea de Medicina si Farmacie Victor Babes din Timisoara, ROMANIA

## Abstract

This study aimed to determine differences in the hip abductor muscle activity during clam exercise at different hip flexion and femoral anteversion angles. Thirty healthy females were divided into two groups based on the femoral anteversion angle: the excessive femoral anteversion group and the normal group. Clam exercise was performed at three different hip flexion angles (60°, 45°, and 30°). Tensor fascia latae, gluteus medius, and superior portion of gluteus maximus activities were measured during the exercise, and the results were normalized to the activity during maximum voluntary isometric contraction to calculate the gluteal-to-tensor fascia latae muscle activation index. The superior portion of gluteus maximus activities at a hip flexion of 60° and 45° were greater than that at 30°. The excessive femoral anteversion group had a lower gluteal-to-tensor fascia latae muscle activation index than the normal group; the gluteal-to-tensor fascia latae muscle activation index for hip flexion at 60° was higher than that at 45°, and the gluteal-to-tensor fascia latae muscle activation index for hip flexion at 60° and 45° were higher than that at 30°. Therefore, the femoral anteversion angle and hip joint position were related to the activity of the hip abductor muscles during clam exercise. These findings may provide a rationale for instructing exercises to maximize the activity of the hip abductor muscles in individuals with an excessive femoral anteversion angle.

## Introduction

Decreased strength and activity of the hip abductor muscles are associated with lower back [1], hip [2,3], and knee [4,5] pain and the risk of sports injuries, such as anterior cruciate ligament injuries, patellofemoral pain, and ankle instability [6–11]. Therefore, exercise training programs have emphasized increasing the hip abductor muscle activity during rehabilitation after sports injuries of the lower extremities and trunk [4,8–18]. Analyzing the muscle activity during exercise and selecting a method that can effectively increase the activity of the hip abductor muscles is important [19]. The tensor fascia latae (TFL), gluteus medius (GMED), and superior portion of the gluteus maximus (SUP-GMAX) are representative hip abductor muscles. The attachments and fiber orientations of these muscles vary, and each muscle plays a distinctive role in lower extremity kinematics and stabilization tasks [20–22].

Weakness of GMED and SUP-GMAX can cause compensatory TFL overactivity during torque exertion of the hip abductors [23,24]. TFL attaches to the iliotibial band, which, in turn, is attached to the lateral patella. Thus, TFL overactivity can cause excessive internal rotation of the hip joint, as well as lateral spin and translation of the patella, via the pulling tension from the iliotibial band [25]. This muscle and tension imbalance has been implicated in the development of patellofemoral joint pain [8,26,27]. Therefore, it is important to identify exercise methods that increase GMED and SUP-GMAX activities with minimal activation of TFL [28–32].

Clam exercise increases gluteal muscle activity, including that of GMED and SUP-GMAX [32], with minimal activation of TFL [28,31]. This exercise is frequently prescribed in rehabilitation and sports settings [14,33]. Changes in the hip angle during clam exercises alter the orientation of the fibers and insertion of the hip abductor muscles, thereby affecting muscular activity [32]. A study analyzing the relationship between the hip flexion position and muscle activity during clam exercise showed that GMED and SUP-GMAX activities were higher with 60° hip flexion than with shallow hip flexion [32]. Therefore, adjusting the hip joint position during clam exercise to maximize gluteal muscle activity is vital [31].

Changes in the femoral anteversion angle alter the fiber orientation and insertion of the hip abductor muscle and influence its activity during clam exercises. Nyland et al. [34] compared the activity of lower extremity muscles during clam exercise in healthy female participants divided into groups according to the femoral anteversion angle. They found that GMED activity was 34% lower in participants with excessive femoral anteversion than in participants with normal femoral anteversion. However, the hip flexion angle during clam exercise was only 45° [34], and activity changes in other hip flexion positions were not analyzed. Therefore, the hip flexion position in clam exercise that maximizes gluteal muscle activities while minimizing TFL activity is unknown for those with an excessive femoral anteversion angle.

An excessive femoral anteversion angle may increase the risk of certain sports injuries, such as knee ligament injuries [35–37], which are frequently observed in females [37]. Excessive femoral anteversion angle causes excessive hip internal rotation and knee valgus [38,39]; thus, it is necessary to identify the hip flexion position that increases GMED and SUP-UGM activity during clam exercises, as they play a crucial role in maintaining the lower extremity alignment [38,39]. Therefore, this study aimed to clarify the relationship between the activity of hip abductor muscles during clam exercise and the femoral anteversion angle and hip flexion position in females. We hypothesized that those with excessive femoral anteversion would have a lower activity of the hip abductor muscles than those with normal femoral anteversion and that this activity would increase with an increased hip flexion angle.

## Materials and methods

### Design

This was a cross-sectional observational study. The activities of TFL, GMED, and SUP-GMAX were measured during clam exercise in three different hip flexion positions in this study to determine whether the activity of the hip abductor muscles is related to the femoral anteversion angle and hip flexion position in healthy females.

### Participants

Thirty healthy females recruited between June 29, 2021 and April 1, 2022 participated in this study. The participants were divided into two groups based on the femoral anteversion angle: the excessive femoral anteversion (EFA) and normal (N) groups. The inclusion criteria were as follows: (1) aged 18–40 years, (2) sports activity once a week or more, and (3) no history of surgery on the lower extremities or trunk. The exclusion criteria were as follows: (1) those who were restricted or prohibited from participating in sports for medical reasons; (2) stopped participating in sports for more than 1 month, 3 months before the measurements; (3) complained of pain in the lower back or lower limbs during the measurements; and (4) those with femoral anteversion angle of 20–30° or <8°. The sample size was calculated using G*power 3.1.9.2 (Heinrich-Heine University, Germany) and the number of participants required for a two-factor (femoral anteversion) × three-factor (exercise condition) two-way ANOVA (repeated measures, within-between interactions) to compare differences in muscle activity (α = 0.05, power = 0.8, partial η2 = 0.06 for effect size) [40]. The required number of participants was estimated to be 28.

### Ethical considerations

All experimental procedures were approved by the Institutional Review Board of Tokyo Medical and Dental University (approval number: M2020-259) and followed the principles of the Declaration of Helsinki for medical research involving human participants. Written informed consent was obtained from all the participants aged over 18 years and also from the parents/legal guardians of participants younger than 18 years.

### Procedure

Dominant-leg activity was measured [41,42]. The dominant-leg was defined as the leg that kicked the ball (right, 28 participants; left, 2 participants). The femoral anteversion angle and maximum voluntary isometric contraction (MVIC) were measured, and electromyography (EMG) was performed using a surface electromyograph. The EMG results were normalized to the MVIC EMG amplitudes, and all measurements were performed on the same day. The measurement order for each exercise was randomized.

### Femoral anteversion angle measurement and group classification

Craig’s test [5] was used to measure the femoral anteversion angle. The participant was placed in a prone position in the intermediate position of the hip, with the knee flexed at 90°. The lower extremity was fixed where the greater trochanter passively touched the most lateral position during hip internal rotation, and the angle of hip internal rotation was measured [5] using manual goniometers. The femoral anteversion angle was measured by taking the average of the two measurements. A single examiner performed all measurements of the femoral anteversion angle. Those with femoral anteversion angles >30° were assigned to the EFA group, while those with angles of 8° to <20° were assigned to the N group [5,43]. A strong positive correlation has been reported between the femoral anteversion angle measured using Craig’s test and that measured using CT in previous studies [44–46]. The inter-rater reliability (95% confidence interval) for measuring the femoral anteversion angle using Craig’s test was reported to be 0.88 (0.68–0.96) [5].

### Surface electromyography

**Fig 1:**
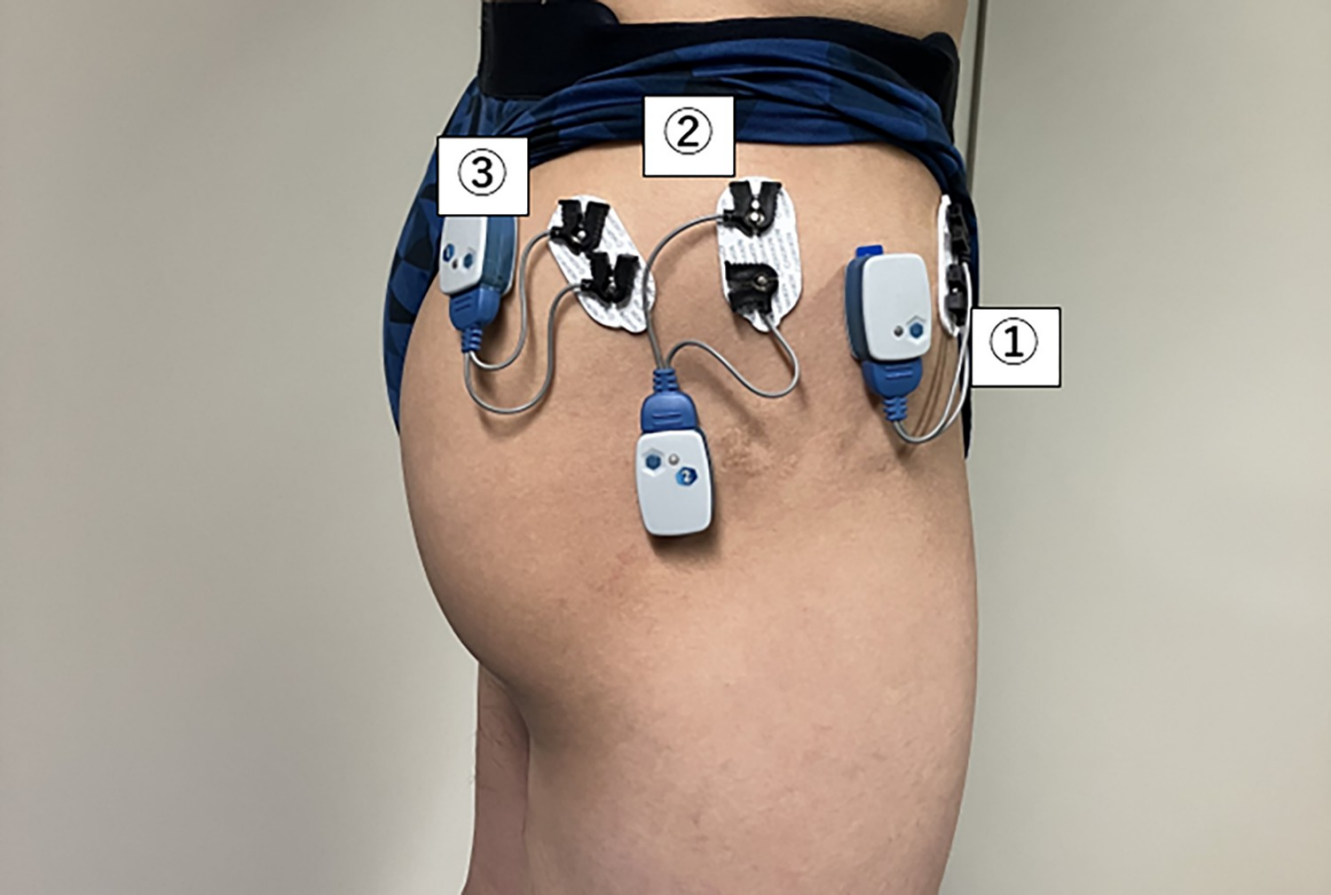
EMG electrode placement. ①: The tensor fascia latae; ②: The gluteus medius; ③: The superior portion of the gluteus maximus.

Surface EMG was performed using Ag/AgCl double-surface electrodes (Noraxon Dual Electrodes, Noraxon USA Inc., Scottsdalte, AZ, USA). Before electrode application, the skin was shaved and washed with isopropyl alcohol to reduce skin impedance [47]. The muscles tested were TFL, GMED, and SUP-GMAX. Electrodes were placed parallel to the muscle. The electrode application site was determined based on previous studies (Table 1 and Fig 1) [48–50]. Visual observation of EMG signals during manual muscle testing was used to assess crosstalk. Crosstalk is affected by the electrode size, position, shape, and distance between electrodes [51], which were minimized by using relatively small electrodes (1.0 cm diameter) and a small distance (2.0 cm) between electrodes. Data were collected using a wireless EMG system (Ultium-EMG; Noraxon USA Inc).

**Table 1:** EMG electrode placement.

Muscle	EMG electrode placement	MVIC positions and resistance sites
**TFL**	2 cm distal to and slightly lateral to the superior anterior iliac spine, parallel to the long axis of the thigh	Hip abduction movement against the resistance of the distal thigh in the side-lying position with the hip in 45° hip flexion and 30° hip abduction in knee extension
**GMED**	Midpoint of the iliac crest and greater trochanter	Hip abduction movement against the resistance of the distal thigh in the side-lying position with hip in 30° hip abduction
**SUP-GMAX**	Midpoint of the second sacral spinous process and the posterior part of the greater trochanter	Hip extension movement against the resistance of the distal posterior thigh in the supine position with 0° knee extension and 10° hip extension

EMG, electromyography; MVIC, maximum voluntary isometric contractions; TFL, tensor fascia latae; GMED, gluteus medius; SUP-GMAX, superior portion of the gluteus maximus.

### Maximum voluntary isometric contractions and reference trials

The EMG amplitude during MVIC was measured for each muscle to normalize the EMG amplitude during the exercise task [52]. The MVIC measurement position was based on previous studies [31,48–50,53,54] that used manual muscle testing for each muscle (Table 1). Following adequate practice, MVIC testing was performed for three sets of 5 s. A 2-min rest period was provided between MVIC measurements to eliminate the effects of induced fatigue [47].

### Exercise tasks and conditions

**Fig 2:**
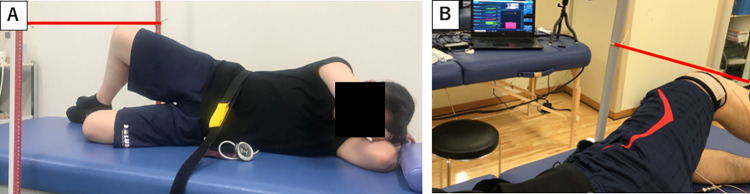
Clam exercises and experimental set-up. A: The clam exercise. B: Torque values during the exercise task are displayed on the monitor; the participants exert 50% of the maximum torque value.

The task was a side-lying clam exercise, with the lumbopelvic girdle in the intermediate position and the knee bent at 90° (Fig 2).

The participants were instructed to maintain the lumbopelvic girdle in the mid-position (with the superior anterior iliac spine facing forward) and the upper (dominant) leg in maximum abduction with external rotation. The exercise consisted of three different hip flexion angles (30°, 45°, and 60°), with the lumbopelvic girdle in the intermediate position. A bar was placed at the knee at maximum hip abduction with external rotation on the measurement side. This was necessary to provide feedback on the exercise position. A manual goniometer was used to check the hip flexion angle for each exercise condition [55,56].

The exercise load was set at 50% of the maximum hip abduction with external rotation torque value for each exercise condition [57]. Before performing the exercise, the maximum hip abduction with external rotation torque value for each exercise condition was measured, and the hip abduction with external rotation torque value at 50% MVIC for each exercise condition was calculated [57]. A hand-held dynamometer was placed on the distal thigh against the direction of motion to measure the hip abduction with external rotation torque during muscle exertion. Participants were instructed to exert a torque value in the range of 50% MVIC ±5%. In front of the participants, a monitor displayed the torque value exerted and the target load in real-time (Fig 2). During the exercise, the participants received visual feedback on the exercise. Three sets of 5-s holds were performed for each exercise condition, with each exercise separated by a 10-min rest period. EMG amplitudes were measured during exercise.

A pressure biofeedback unit (Stabilizer, Chattanooga Group Inc., USA) was placed in the lumbar region to assess the lumbar pelvic girdle stability during the task. The pressure biofeedback unit scale was maintained at 35–45 mmHg during the task [14,58,59].

### Data processing

The EMG amplitude of each muscle was measured using a surface EMG (Ultium EMG, EM-U810M8, Tele Myo2400, Noraxon USA Inc). The EMG amplitudes were band-pass filtered at 20–500 Hz and sampled at 2,000 Hz on a personal computer using a receiver. The amplitude of each EMG during each task was the root mean square (RMS) processed in moving windows of 50 ms, and the EMG amplitudes were full-wave, rectified, and smoothed.

The RMS value during each task was defined as the average of the values for the 1.0-s intervals exhibiting the maximum stability and maximum value within 5 s of the exercise task. The RMS values during MVIC were the maximum values among the extracted 1.0-s. The RMS value for each exercise condition was averaged over three trials. Each RMS value was normalized to that obtained during MVIC and used as the muscle activity (%).

The gluteal-to-TFL muscle activation (GTA) index was calculated from the following equation based on previous studies [31]: {[(GMED/TFL) × GMED] + [(SUP-GMAX/TFL) × SUP-GMAX]}/2 (%). The GTA index (%) was calculated by inputting the normalized RMS values during each exercise task in each muscle into the formula. Higher GTA index values indicate higher GMED and SUP-GMAX activities, compared to the TFL activity [31]. These data were anonymized and no individual participant information was accessed during or after data collection.

### Statistical analysis

The normality of the distribution of each variable was determined using histograms and the Shapiro–Wilk normality test. For each variable, descriptive statistics are presented as mean ± standard deviation for normally distributed variables and median (interquartile range) for non-normally distributed variables.

Unpaired t-tests and Mann–Whitney U tests were used to analyze differences in demographic variables between the groups. An analysis of variance for a split-plot factorial design was used to analyze differences in the normalized EMG amplitude and GTA index for each group (femoral anteversion angle: EFA group, N group) and exercise condition (hip flexion at 60°, 45°, 30°). Where the main effects and significant interactions were found (p < 0.05), the Bonferroni post-hoc test and unpaired t-test were performed. Cohen’s d was used to calculate the effect size for the comparison between the exercise tasks. Cohen’s d values of 0.20–0.49, 0.50–0.79, and ≥ 0.80 corresponded to small, medium, and large effects, respectively [60]. The significance level was set at 5%. Statistical analyses were performed using IBM SPSS statistics version 27 (IBM Corp., Armonk, NY, USA).

## Results

### Demographics of each group

**Fig 3:**
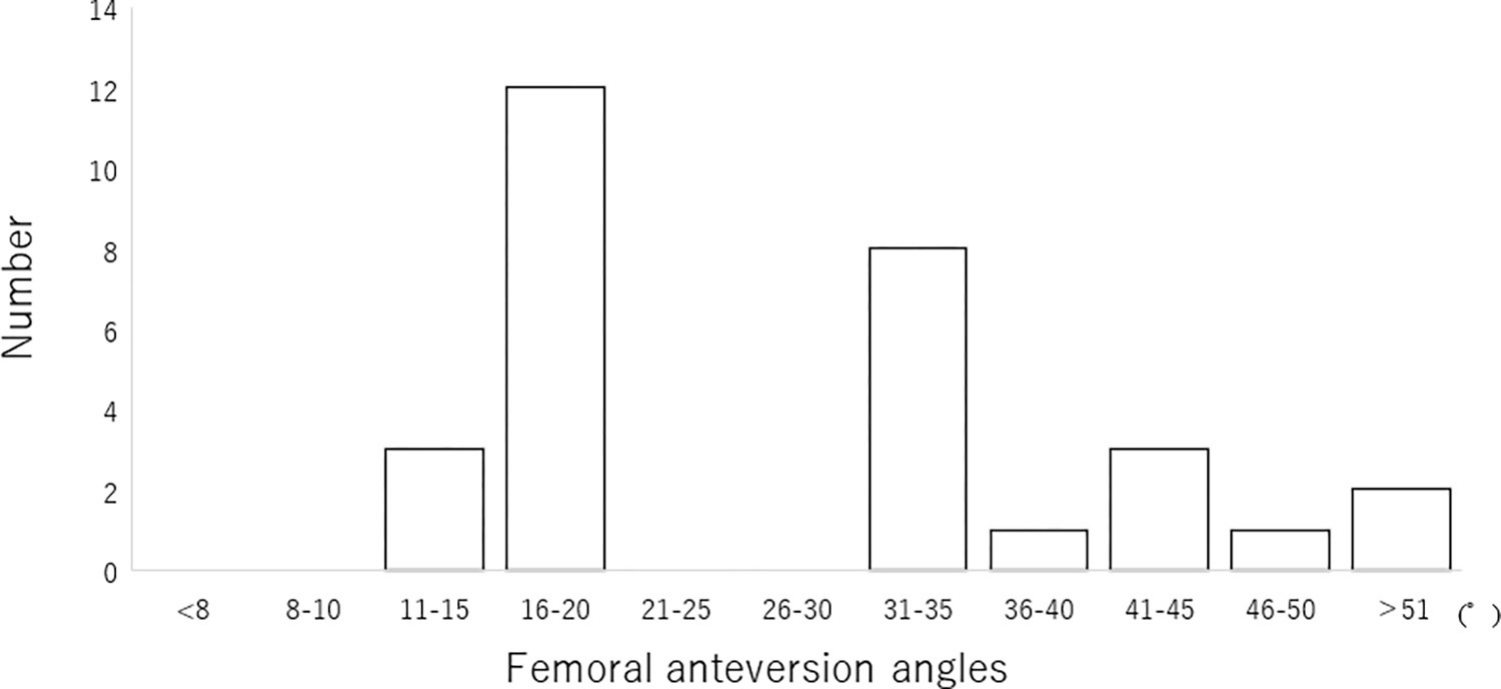
Distribution of femoral anteversion angles.

Participants were divided into two groups. The femoral anteversion angles of the participants showed a bimodal distribution (Fig 3). The Shapiro–Wilk normality test revealed non-normal distribution of age and the femoral anteversion angle. The data of the remaining attributes were normally distributed. The Mann–Whitney test conducted to analyze the differences in age and the femoral anteversion angle between the groups revealed differences in the femoral anteversion angle (p<0.001, Table 2). No differences were observed between the groups in terms of height, weight, or BMI (Table 2).

**Table 2:** Comparison of demographics between groups (N = 30)^α^.

	All	EFA group (n = 15)	N group (n = 15)	p-Value
**Age** ^**β**^ **, y**	26.0 (4.3)	27.0 (2.0)	26.4 (6.0)	0.531
**Height, cm**	159.3±4.8	158.6±4.2	160.0±5.4	0.456
**Weight, kg**	51.1±4.9	51.6±5.6	50.7±4.4	0.614
**Body mass index, kg/m** ^**2**^	20.1±1.6	20.5±1.8	19.8±1.3	0.240
**Femoral anteversion angle** ^**β**^ **, °**	25.0 (17.8)	38.3 (9.6)	19.7 (3.8)	0.000

^α^ Data are reported as mean± standard deviation or median (interquartile range).

^β^ Differences between groups were analyzed using the Mann–Whitney test.

EFA: Excessive femoral anteversion, N: Normal.

### Normalized EMG amplitude

For TFL and GMED activities, the split-plot analysis of variance revealed no main effects or interactions between factors (Tables 3 and 4). There was no interaction between the factors for the SUP-GMAX activity; nevertheless, there was a main effect of the exercise condition (p<0.001, Table 5). Post hoc tests revealed that the hip flexions at 60° and 45° were significantly higher than that at 30° (p<0.001, Cohen’s d:0.57 and p = 0.011, Cohen’s d: 0.34, respectively; Table 5).

**Table 3:** Differences in the activity of TFL for each exercise condition in each group.

Exercise condition	Activity			Main effect		Interaction
	EFA group	N group	All	Group	Exercise condition	
**Flexion at 60°**	15.2±8.8	18.1±12.0	16.7±10.3	0.905	0.652	0.106
**Flexion at 45°**	18.6±13.4	16.6±11.2	17.6±12.0			
**Flexion at 30°**	18.3±12.3	15.9±10.6	17.1±11.1			
**All**	17.4±11.5	16.9±11.0				

TFL, tensor fascia latae; EFA, excessive femoral anteversion; N, normal.

**Table 4:** Differences in the activity of GMED for each exercise condition in each group.

Exercise condition	Activity			Main effect		Interaction
	EFA group	N group	All	Group	Exercise condition	
**Flexion at 60°**	13.0±5.5	17.8±12.5	15.4±9.6	0.297	0.193	0.345
**Flexion at 45°**	13.1±5.8	15.9±9.5	14.5±7.7			
**Flexion at 30°**	12.5±6.6	14.5±9.2	13.5±7.8			
**All**	12.9±5.9	16.1±10.4				

GMED, gluteus medius; EFA, excessive femoral anteversion; N, normal.

**Table 5:** Differences in the activity of SUP-GMAX for each exercise condition in each group.

Exercise condition	Activity			Main effect		Interaction
	EFA group	N group	All	Group	Exercise condition	
**Flexion at 60°**	38.1±13.8	49.7±22.0	43.9±18.6^*^	0.184	0.000	0.552
**Flexion at 45°**	35.2±16.2	45.0±25.3	40.1±21.1^†^			
**Flexion at 30°**	29.7±15.7	36.5±23.4	33.1±19.6			
**All**	34.4±15.2	43.7±23.5				

*: p <0.001 (Compared to Hip flexion 30°)

†:p = 0.011(Compared to Hip flexion 30°)

SUP-GMAX, superior portion of the gluteus maximus; EFA, excessive femoral anteversion; N, normal.

### GTA index

There was no interaction, whereas there was a main effect of factors for femoral anteversion (p = 0.044) and the exercise condition (p<0.001, Table 6). A post hoc test revealed that the GTA index of the EFA group was significantly lower than that of the N group (p = 0.044, Cohen’s d: 0.68, Table 6). In the exercise condition, 60° of hip flexion was significantly higher than 45° (p = 0.042, Cohen’s d: 0.36), while 60° and 45° of hip flexion were significantly higher than 30° (p<0.001, Cohen’s d:0.69 and p = 0.022, Cohen’s d:0.39, respectively; Table 6).

**Table 6:** Differences in the GTA index for each exercise condition in each group.

Exercise condition	GTA index			Main effect		Interaction
	EFA group	N group	All	Group	Exercise condition	
**Flexion at 60°**	76.5±46.4	117.8±89.7	97.2±72.0§†	0.044	0.000	0.942
**Flexion at 45°**	54.0±43.6	95.0±58.6	74.5±53.9‡			
**Flexion at 30°**	36.7±22.4	73.0±60.3	54.9±47.5			
**All**	55.8±41.5*	95.3±71.8				

*: p = 0.044 (compared to the N group)

§: p = 0.042 (Compared to Hip flexion 45°)

† p <0.001　(Compared to Hip flexion 30°)

‡: p = 0.022 (Compared to Hip flexion 30°).

GTA, gluteal-to-tensor fascia latae muscle activation; EFA, excessive femoral anteversion; N, normal.

## Discussion

In this study, SUP-GMAX activity during clam exercise was higher when the hip flexion angle was increased. In addition, the GTA index was higher under these conditions. Individuals with excessive femoral anteversion angles had lower GTA indices than those with normal anteversion angles, regardless of the hip flexion angle. The findings of the present study partially supported our hypothesis.

We found that the SUP-GMAX activity was significantly higher at 60° and 45° of hip flexion than at 30°. Willcox et al. [32] analyzed the activity of the gluteus maximus during clam exercise in healthy participants and reported that the gluteus maximus activity at 60° hip flexion was higher than that at 0° hip flexion. Our findings are consistent with those of previous studies. Muscle activity increases when the axis of motion coincides with the orientation of the muscle fibers [61]. Therefore, as the hip flexion angle increases, the SUP-GMAX activity increases as the orientation of the fibers of SUP-GMAX approximates that of the femur, the axis of motion of the hip joint.

The GTA index was significantly higher at 60° and 45° of hip flexion than at 30° and significantly higher at 60° than at 45°. There was no difference in activity between the exercise tasks, and the SUP-GMAX activity increased as the hip flexion angle increased. Willcox et al. [32] observed increased gluteal muscle activity during clam exercise in a position with an increased hip flexion angle, while the TFL activity remained unchanged. The results of this study supported those of previous studies. The GTA index was increased in this study, due to the increased SUP-GMAX activity relative to the TFL activity as the hip flexion angle increased. The orientation of the SUP-GMAX fibers approximates the axis of motion when the hip flexion angle increases, favoring the direction of hip abduction/external rotation motion. This may have resulted in higher muscle activity. Thus, the findings of this study provide electromyographic evidence for the anatomical muscle orientation characteristics of SUP-GMAX. These findings suggest that it is important to perform the position with an increased hip flexion angle to increase the ratio of the SUP-GMAX activity to TFL activity during the clam exercise.

The GTA index of the EFA group was significantly lower than that of the N group. No previous studies have assessed the relationship between the GTA index and the femoral anteversion angle during clam exercise. In the mid-position of the hip, if the femoral anteversion angle is excessive, the position of the greater trochanter is displaced posteriorly compared to that of the normal anteversion angle [62,63]. When the femoral anteversion angle is increased, the arm length in hip extension and abduction is reduced [63,64]. The SUP-GMAX length is shortened when the anterior femoral anteversion angle is excessive [65]. The muscle activity varies with the muscle length; shortening the muscle length reduces the muscle activity [66,67]. Thus, the SUP-GMAX activity of females with excessive femoral anteversion is lesser than that of those with normal femoral anteversion, suggesting that it is difficult to maximize SUP-GMAX activity regardless of the hip flexion angle.

No significant differences in GMED activity during clam exercise based on the femoral anteversion angle were observed in this study. The activity of the GMED during clam exercise was lower in the group with a larger femoral anteversion angle in a study that analyzed differences in GMED activity during clam exercise based on femoral anteversion in two groups of healthy female participants [34]. Therefore, the findings of this study contradict the findings of previous studies. Nyland et al. [34] defined the maximum angle of hip internal rotation in the prone position as the femoral anteversion angle, with the cut-off value being the median value for all participants. GMED activity was analyzed as the ratio of TFL and GMAX activities. The participants, grouping method and analysis method of the previous study were different from those of this study; hence, this could be why our findings contradict those of previous studies.

GMED and TFL activities were not affected by the hip angle during clam exercise in this study. Wilcox et al. [32] analyzed TFL and GMED activities during clam exercise at different hip flexion angles and reported that different hip flexion angles had no effect on TFL activity; however, GMED activity increased with increasing hip flexion angle [32]. TFL activity was not affected by the differences in hip angle in the present study. This finding is consistent with those of the study by Wilcox et al. [32]. However, the GMED activity observed in this study differed from that reported by Wilcox et al. [32]. The hip flexion angles were set at 0°, 30°, and 60° in the study by Wilcox et al. [32]. In contrast, the hip flexion angles were set at 30°, 45°, and 60° in the present study. Moreover, the hip flexion angle settings were more detailed than those reported in previous studies [32]. Consequently, the differences between the present study and previous studies [32] in terms of the hip flexion angles may have led to the differences in GMED activity. GMED is divided into anterior, middle, and posterior fibers based on anatomical structural features, and these fibers perform different actions [61,68]. Moore et al. [69] measured the activities in three segments of GMED (anterior, middle, and posterior) during clam exercise and reported that the activity in the posterior segment was higher than those in the anterior and middle segments. Morphologically, the anterior and middle segments have less favorable moment arms in the transverse plane for external rotation than the posterior segment. Thus, it is important to analyze the different segments of GMED separately [50]. The electrode was attached to the middle segment of GMED in this study. Thus, it may be necessary to divide the GMED activity into different categories corresponding to the activity of each segment and analyze the activity characteristics according to the femoral anteversion angle and hip flexion angle in detail.

Based on these findings, to effectively increase the gluteal muscle to TFL activity ratio, the hip joint should be flexed at 60°, regardless of the femoral anteversion angle. The GTA index in the EFA group was significantly lower than that in the N group, regardless of the flexion angle. Additional exercises must be implemented in females with excessive femoral anteversion angles to increase the activity of the gluteal muscles. Therefore, this finding may provide a basis for designing exercises to effectively increase the hip abduction muscle strength while considering the femoral anteversion angle of the participants.

### Limitations

This study had some limitations. First, measures were taken to reduce crosstalk as much as possible; however, surface EMG measurements may have still been affected by crosstalk [70]. In addition, although none of the participants in this study were classified as obese based on their BMI values, muscle activity was measured at the hip, which has a high distribution of adipose tissue in women. Second, the primary findings were only based on the muscle activity. It is impossible to draw conclusions regarding the effects of the results on sports injury risks or training. Third, the GTA indices analyzed in this study were for exercises on open kinetic chains and cannot be generalized to exercises on closed kinetic chains or clam exercises without resistance or with elastic bands. Fourth, this study analyzed activity during short isometric exercises. Thus, it is unclear whether a change in activation is observed during fatigue. Finally, as this study only included healthy females, sex-based differences remain unclear. The results may differ between males and those with back or hip pain. Therefore, it will be necessary to increase the range of participants and exercise load conditions. Future studies should examine the relationship between performance variables and the effects of training.

## Conclusion

SUP-GMAX activity was higher in the clam exercise condition with an increased hip flexion angle. Additionally, the GTA index was higher under these conditions. Those with an excessive femoral anteversion angle had a lower GTA index than those with a normal anteversion angle, regardless of hip flexion angle. The findings revealed that factors including anterior femoral anteversion and hip flexion angle were related to the amount of hip abductor muscle activity during clam exercise. These findings may provide a rationale for instructing exercises to maximize hip abductor muscle activity in individuals with an excessive femoral anteversion angle.

## Supporting information

S1 Data (XLSX)
